# Mechanisms of Zuogui Pill in Treating Osteoporosis: Perspective from Bone Marrow Mesenchymal Stem Cells

**DOI:** 10.1155/2018/3717391

**Published:** 2018-09-19

**Authors:** Aofei Yang, Chaochao Yu, Fang You, Chengjian He, Zhanghua Li

**Affiliations:** ^1^Department of Orthopedics, Hubei Provincial Hospital of TCM, Wuhan 430061, China; ^2^Hubei Province Academy of Traditional Chinese Medicine, Wuhan 430074, China; ^3^Hubei University of Chinese Medicine, Wuhan 430061, China; ^4^The Second Clinical College, Guizhou University of Chinese Medicine, Guiyang 550000, China; ^5^Tongren Hospital of Wuhan University, Wuhan 430060, China

## Abstract

The current treatment strategies for osteoporosis (OP) involve promoting osteogenic differentiation and inhibiting adipogenic differentiation of bone marrow mesenchymal stem cells (BMSCs). According to a theory of traditional Chinese medicine (TCM), the kidneys contain an “essence” that regulate bone metabolism and generate marrow. Kidney disorders are therefore considered to be a major cause of OP as per the principles of TCM, which recommends kidney-tonifying treatments for OP. The Zuogui pill (ZGP) is a classic kidney-tonifying medication that effectively improves OP symptoms. Studies have shown that ZGP can promote the osteogenic differentiation of BMSCs, providing scientific evidence for the TCM theory linking kidneys with bone metabolism. In this review, we have provided an overview of recent studies that examined the underlying mechanisms of ZGP mediated regulation of BMSC osteogenic and adipogenic differentiation.

## 1. Introduction

Osteoporosis (OP) is a common metabolic bone disease characterized by decreased bone mass and density and abnormal microstructure, all of which result in increased bone fragility. OP is broadly divided into primary and secondary OP, and the former is further classified into postmenopausal, senile, and idiopathic forms [1, 2]. The incidence of OP is increasing annually with the aging of the global population [2]. A steady-state imbalance between bone resorption and bone formation is the pathological basis of OP [1]. As the mechanism of osteoclast differentiation became clearer, several anti-bone resorption medications such as bisphosphonates began to emerge as promising therapies for OP. However, long-term use of osteoclast inhibitors can inhibit bone remodeling and result in various side-effects [3], including mandibular necrosis [4]. Therefore, greater attention has now been directed towards understanding the mechanisms involved in promoting bone formation.

Bone marrow mesenchymal stem cells (BMSCs) were identified nearly half a century ago and have since been extensively studied. BMSCs can differentiate under specific conditions into multiple cells, including osteoblasts, chondrocytes, cardiomyocyte-like cells, vascular endothelial cells, neurons, hepatocytes, and adipocytes [5]. As a result of their multipotent differentiation and self-renewal capabilities, the ease of their isolation and* in vitro* expansion, and low risk of immune rejection, BMSCs are highly promising in stem cell based therapies [6]. Several studies have found that reduced osteogenic differentiation and increased adipogenic differentiation of BMSCs are the key pathogenic drivers of OP [7–9]. Therefore, blocking the adipogenic differentiation and promoting osteogenic differentiation of BMSCs is a potential strategy to restore the imbalanced bone metabolism in OP [9–11].

According to TCM theories, a kidney “essence” controls bone metabolism and generates marrow; if this essence is sufficient, bone marrow will be engorged, and the bone will be healthy and strong. The deficiency of this essence is the major pathological cause of OP as per TCM principles. Previous studies have demonstrated that kidney essence deficiency is closely associated with OP [12], whose onset is most pronounced in patients with kidney disorders [13]. In addition, studies on the quality of life and TCM syndrome of postmenopausal OP patients showed that kidney deficiency occurred in up to 84.7% of the patients [14]. Kidney-tonifying herbal medicines improved bone structure in OP rats by increasing serum estradiol level and bone density [15]. A clinical study by Liu et al. also showed that a kidney-tonifying herbal medicine can increase bone density, lower bone turnover rate, alleviate OP-induced osteopathic pain, and improve the quality of life of patients [16]. These findings all demonstrate that kidney deficiency is closely associated with OP and that kidney-tonifying therapy can alleviate OP [17].

BMSCs are pluripotent adult stem cells that are mainly found in the human bone marrow. They can undergo osteogenesis under certain conditions and are part of the essence and marrow described in TCM. Studies have found that the mechanisms of BMSC proliferation, differentiation, and development are very similar to the role that the kidney essence plays in the growth, development, and aging of an individual [18]. In fact, the primary functions of kidney essence are reflected in the proliferation and differentiation of BMSCs [19]. Recent studies have shown that BMSCs differentiation is a complex process that can be influenced by the synergistic action of multiple factors, including intracellular and extracellular signal transduction, physical and chemical factors, epigenetic modification, transcriptional regulation, and posttranscriptional regulation. However, whether BMSCs differentiation is determined by specific molecules or pathways is currently unclear. On the other hand, kidney-tonifying Chinese herbal medicines have demonstrated multipathway, multifunctional, and multitarget modulatory effects in the prevention and treatment of OP. Therefore, understanding the effect of these medicines on the osteogenic differentiation of BMSCs will provide new insights into the scientific basis of the TCM theory of kidney essence in bone metabolism and OP. The overall effects of classic Chinese kidney-tonifying recipes on BMSC differentiation are currently ill-defined as the previous studies were largely focused on the effect of a single herbal medicine or monomer, which can neither fully reflect the “kidney-tonifying, essence-generating, and marrow-benefiting” properties of these TCM recipes nor the compatibility of these formulations. Although a single herbal medicine is composed of only its chemical constituents or active ingredients, the same medicine will have different pharmacodynamics in different recipes and, under different pathological states, and the resulting effective substance it releases will also be different [20]. At present, Zuogui pill (ZGP) is the best known kidney-tonifying recipe that has been shown to regulate BMSC osteogenic differentiation. In this review, we have provided an overview of current findings on ZGP-induced BMSC osteogenic differentiation and its application in the treatment of OP.

## 2. Signal Transduction Pathways That Regulate BMSCs Differentiation

**Figure 1:**
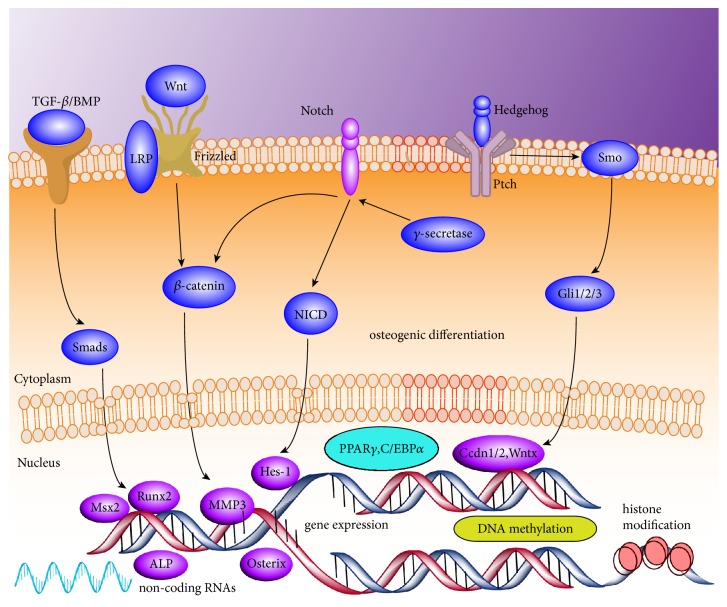
Signal transduction pathways that regulate BMSCs differentiation. TGF-*β*/BMP, Wnt/*β*-catenin, Notch, and Hedgehog signaling pathways are involved in the regulation of BMSC mediated osteogenesis. DNA methylation, histone modification, and noncoding RNAs regulation play a significant role in gene expression associated with osteogenic differentiation of BMSC.

Several signaling pathways are involved in the regulation of BMSC mediated osteogenesis and adipogenesis, and their mechanisms have been previously reported in numerous reviews [21–24]. These pathways include those of bone morphogenic protein (BMP)/Smad, Wnt, Hedgehog, Notch and fibroblast growth factor signaling, in addition to epigenetic regulation (Figure 1). It is apparent that BMSC differentiation is a multistep, multitarget, and multifactorial process, but whether this process is regulated by specific key molecules or pathways is currently unclear. Therefore, finding specific targets is the top concern for the treatment of OP.

## 3. Role of ZGP in the Regulation of BMSCs Osteogenic Differentiation

ZGP is a well-known classic kidney-tonifying recipe that originated from “The Complete Works of Jing-yue” by Zhang Jingyue in Ming dynasty. This formulation consists of processed rehmannia root,* Rhizome dioscoreae*, goji,* Cyathula officinalis*,* Cornus officinalis*,* Cuscuta chinensis*, deer horn gelatin, and tortoise plastron gelatin and generates the kidney essence which benefits marrow and strengthens the muscles and bones. ZGP has been clinically proven to significantly increase the bone density of primary OP patients [25]. Animal studies have also demonstrated that ZGP and Yougui pill (YGP) can prevent and treat bone loss in the ovariectomized rat model of OP by increasing bone density and trabecular bone surface area, restoring serum levels of bone metabolism markers, and improving bone metabolism [26–28]. Analysis of ZGP constituents by ultrahigh performance liquid chromatography-mass spectrometry (UHPLC-MS) revealed indole acetaldehyde, retinylester, and alpha-CEHE as the potential active ingredients, strongly suggesting that the anti-OP mechanisms of ZGP may be associated with the tryptophan and retinol metabolism pathways [29]. Indole acetaldehyde can reversibly convert to tryptophan, and the latter is a precursor of 5-hydroxytrptamine which is closely associated with bone formation and resorption [30]. Similarly, retinyl ester can reversibly convert to vitamin A [31], and the latter is present in osteoclasts and osteoblasts. Excessive levels of vitamin A can inhibit osteoblast activity and enhance osteoclast activity [32]. Alpha-CEHE is a metabolite of vitamin E [33], and vitamin E level is closely associated with osteoclast activity [34] and pathogenesis of OP [35].

The morphology and the proliferative and differentiation capabilities of BMSCs decline with age [36]. Senile BMSCs are larger and more flattened and have enlarged nuclei and fewer mitochondria that often appear swollen, expanded endoplasmic reticulum and lipofuscin deposition. Furthermore, the proportion of polygonal and star-shaped BMSCs increases with age while that of fibrous spindle-like BMSCs decreases [37]. Therefore, enhancing the proliferative capability and improving the senile morphology of BMSCs can induce their osteogenic differentiation [38]. Studies have shown that ZGP can reduce ultrastructural damage and maintain the normal spindle-like morphology of BMSCs in aged rats [39, 40], promote proliferation [41], and inhibit apoptosis as indicated by elevated caspase-3 and decreased Bcl-2 levels [42, 43].

Alkaline phosphatase (ALP) is an early marker of osteoblast differentiation. ALP levels begin to increase during matrix synthesis and peak at the calcification phase [44]. It is also a marker for osteoblast maturation and can reflect the ability of osteoblast to synthesize type I collagen and form the bone matrix [45]. Therefore, ALP can be used as a key indicator of the functional status of osteoblasts to assess the degree of osteogenic differentiation [46]. OP patients not only have decreased BMSC proliferation and osteogenic differentiation capability, but also increased adipogenesis, reduced levels of type I collagen, and reduced calcified nodule formation, all of which culminate in decreased number of osteoblasts and increased number of osteoclasts [36]. Calcified nodules are the products of the calcium salts secreted by osteoblasts and are one of the key markers of osteoblast differentiation and maturation [47]. One study reported that ZGP-containing serum (ZGP serum) could significantly promote the formation of mineralized nodules [48] and increase the expression of ALP and type I collagen [49, 50], thereby inducing the osteogenic differentiation of BMSCs [51, 52]. In addition, ZGP serum can inhibit BMSC adipogenic differentiation in ovariectomized rats by inhibiting the expression of peroxisome proliferator-activated receptor *γ* (PPAR*γ*), lipoprotein lipase (LPL), and fatty acid binding protein 4 (FABP4) [53], which are key regulators of adipogenesis [54].

Further studies have shown that the osteogenic differentiation-promoting effect of ZGP serum on BMSCs is associated with the upregulation of *β*-catenin and LRP-5 protein levels [55]. In the canonical Wnt/*β*-catenin signaling pathway, binding of Wnt to its receptor Frizzled and related receptor LRP5/6 leads to cytosolic accumulation of *β*-catenin. Upon nuclear translocation, *β*-catenin acts on the transcription factors Tcf/Lef to activate several downstream genes including matrix metalloproteinase-3 (MMP-3), which subsequently promote BMSC osteogenic differentiation and inhibit their adipogenic differentiation [56]. A reduction in LRP-5 activity results in OP [57]. On the other hand, the p38 MAPK signaling [58] and TGF-*β*1/Smad signaling pathways [29, 59, 60] are also involved in ZGP serum-induced BMSC osteogenic differentiation. In addition, ZGP serum can also upregulate the levels of neuropeptides and neurotrophic factors, such as CGRP, SP, VIP, and NPY in BMSCs and bone tissues of ovariectomized OP rats, demonstrating that ZGP serum regulates bone metabolism via the neuroendocrine axis [61].

Chinese medicine compounds consist of complex ingredients that can act on multiple targets and achieve systemic modulatory effects. Therefore, focusing on only a few key genes or proteins may not fully reveal the therapeutic mechanisms of these compounds. Studies using high-throughput gene expression profiling and bioinformatics mining techniques have revealed that* Ttpa*,* Sema3d*,* Lrp2*,* Slc22a5*, and* Plac1* are the direct target genes of ZGP in postmenopausal OP [62]. These genes are involved in the regulation of vitamin E metabolism [63], osteoblast and osteoclast activity [64, 65], and estrogen receptor activity [66], which altogether can affect bone metabolism. Furthermore, ZGP induces a different temporal gene expression profile in BMSCs of ovariectomized rats, primarily characterized by an early downregulation of* Ppig, Rb1cc1*, and* IL-6*. ZGP in fact regulates more genes compared to estradiol valerate [67].* Ppig* encodes peptidylprolyl isomerase G that catalyzes the cis-trans isomerization of peptidylprolyl and is primarily involved in the regulation of cell cycle and cell proliferation [68].* Rb1cc1* encodes the RB1-inducible coiled-coil protein 1 that regulates lysosomal formation [69]. Finally, IL-6 promotes osteoclast activity and enhances bone resorption via the RANKL-RANK-OPGA pathway [70]. These findings demonstrate that ZGP can influence cell proliferation, autophagy, signal transduction, and cell differentiation to extensively regulate BMSCs gene expression and ultimately reduce bone loss.

**Figure 2:**
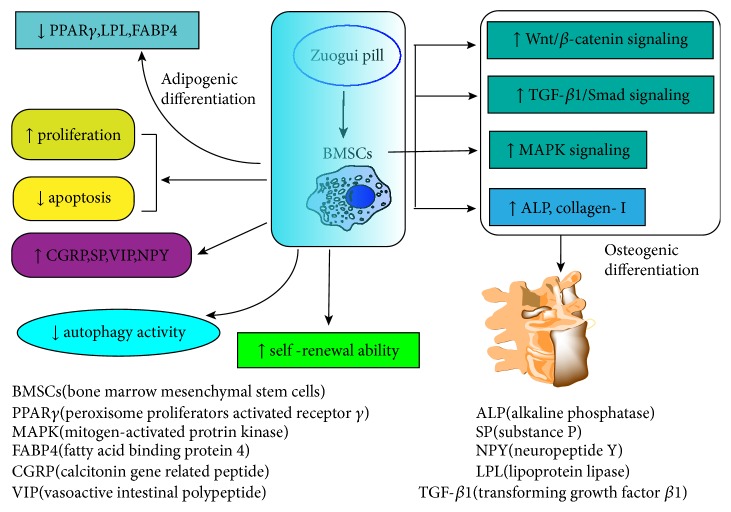
The proosteogenic effects of kidney-tonifying ZGP on BMSCs are mediated through multiple signaling transduction pathways, including upregulation of MAPK, Wnt/*β*-catenin, and TGF-*β*1/Smad signaling pathways. ZGP can also decrease autophagy activity, promote proliferation, and inhibit apoptosis of BMSCs, strengthening the self-renewal capabilities. ↑: upregulate; ↓: downregulate.

In summary, the proosteogenic effects of kidney-tonifying ZGP on BMSCs are mediated through multiple targets and pathways (Figure 2). These results provide scientific evidence for the “kidney dominates bone” theory of TCM and the clinical application of kidney-tonifying recipe for the prevention and treatment of OP.

## 4. Future Directions?

BMSCs have been the main focus of OP pathogenesis and treatment research in recent years. Given that the kidney essence described by TCM shares many similarities with the origin and functions of BMSCs, it is hypothesized that regulation of BMSC osteogenic differentiation may partly be the scientific basis of the kidney-tonifying recipes used in OP treatment. Studies have shown that BMSC differentiation is a complex process and controlled at different stages by various key factors during OP pathogenesis. Therefore, precise regulation of BMSCs will be the next step in OP prevention and treatment. ZGP is a representative kidney-tonifying compound that promotes BMSC proliferation and differentiation into osteoblasts. However, whether the effect of ZGP depends on the stage of BMSC differentiation and whether that would influence the outcome of osteogenic differentiation is still unclear. Therefore, understanding the time and dose effects of ZGP on BMSC osteogenic differentiation will be clinically relevant in the treatment of OP. Although the effect of classic kidney-tonifying compounds on BMSC osteogenic differentiation is still poorly understood, a few preparations such as six-ingredient rehmannia pill, sexoton pill [71, 72], Erxian decoction [73], and Qing'e pill [74] have been shown to promote osteogenic differentiation and inhibit angiogenic differentiation of BMSCs. Therefore, it is important to compare the effects of the different kidney-tonifying compounds on BMSC osteogenic differentiation vis-à-vis OP treatment.

In addition, classic kidney-tonifying formulations can be further classified into the* yin* or* yang* promoting compounds; whether these different formulations have different mechanisms and whether that influence osteogenic differentiation will need to be further studied. Most existing studies on kidney-tonifying compounds focus on their osteogenic differentiation-promoting effects, but their effects on BMSC migration, homing, and adipogenic differentiation are less well understood. In fact, enhancement of BMSC migration and homing and the inhibition of BMSC adipogenic differentiation may also be one of the mechanisms by which kidney-tonifying compounds prevent and treat OP. What is more, osteoporosis is closely related to the balance of osteoblasts and osteoclasts. Current studies are mainly focused on the effects of ZGP on proosteogenic effects; effects on osteoclast involved in bone resorption are less understood. Several studies indicated that mechanisms of ZGP in inhibiting osteoclast bioactivity are associated with upregulating estrogen levels, decreasing inflammatory factors TNF-*α* and IL-17 that correlated with osteoclast activity [75], and downregulating NFAT2 expression [76]. More focus should be given on the regulation of ZGP on osteoclasts in future studies. Since epigenetic modification plays an important regulatory role in stem cell differentiation, understanding the epigenetics of kidney-tonifying preparations in the induction of BMSC osteogenic differentiation will help further elucidate the scientific basis of these compounds in OP treatment. In addition, serum pharmacology-based metabolomics will also help us elucidate the effective constituents of kidney-tonifying compounds that are involved in the regulation of BMSC osteogenic differentiation.
